# Methodological Considerations and Future Directions in Mental Health Multimorbidity Research: Response to Andreeva et al.

**DOI:** 10.3389/ijph.2024.1608114

**Published:** 2025-01-14

**Authors:** Mengqin Zhang

**Affiliations:** Duke Global Health Institute (DGHI), Duke University, Durham, NC, United States

**Keywords:** mental health multimorbidity, anxiety, insomnia, eating disorders, methodological considerations

Dear Editor,

I read with great interest the article by Andreeva et al. [1] on mental multimorbidity patterns among general-population adults, focusing on anxiety, insomnia, and eating disorders. The study’s focus on mental health multimorbidity is particularly relevant, especially as there is growing recognition of the impact of mental health on public health outcomes and healthcare utilization.

The authors provide valuable insights into mental multimorbidity’s prevalence and sociodemographic profiles; however, several methodological aspects merit careful consideration. Of primary concern is the study’s reliance on self-reported questionnaires, particularly the SCOFF questionnaire for eating disorders. Despite SCOFF’s validated screening capabilities, its inability to distinguish between different types of eating disorders poses significant interpretative challenges. This limitation becomes especially critical when considering that different eating disorders exhibit distinct relationships with anxiety and insomnia. Indeed, Levinson et al. [2] have demonstrated that specific eating disorder subtypes show varying patterns of comorbidity with anxiety disorders, highlighting the need for more nuanced diagnostic approaches.

Building on these methodological considerations, the authors' use of sex-specific cutoffs for the STAI-T raises additional questions that require further exploration. While the authors cite previous epidemiological research to support this approach, the impact of these differential thresholds on reported sex differences in anxiety prevalence warrants deeper examination. This concern is particularly relevant in light of [3] research, which reveals how measurement bias in anxiety assessments can substantially influence observed gender differences.

Another crucial aspect requiring attention is the study’s cross-sectional design. Although the authors acknowledge this limitation, its implications extend beyond mere temporal constraints. The inability to establish the chronological sequence of these mental health conditions significantly impacts our understanding of their interrelationships. This point is underscored by [4] longitudinal research, which demonstrates how the temporal ordering of mental health conditions can critically affect treatment outcomes and prognosis.

Furthermore, while the authors consider socioeconomic factors through education and occupation variables, there remains an opportunity for deeper analysis. A more comprehensive examination of how socioeconomic status mediates or moderates the relationships among these mental health conditions could yield valuable insights for developing targeted public health interventions.

Looking ahead, several key directions emerge for future research. Priority should be given to longitudinal studies that can establish temporal relationships among these conditions. Additionally, enhancing the methodology through objective sleep measures, such as actigraphy, alongside self-reported insomnia symptoms would strengthen the findings considerably. The integration of biological markers and genetic data could further illuminate the shared mechanisms underlying these conditions.

While this study makes a significant contribution to our understanding of mental health multimorbidity, addressing these methodological considerations and expanding the theoretical framework would advance the field substantially. The findings ultimately emphasize the critical need for integrated approaches to mental healthcare that acknowledge and address the interconnected nature of these conditions.

Sincerely,

Mengqin Zhang.
